# Supplementary material for: Mechanisms governing the pioneering and redistribution capabilities of the non-classical pioneer PU.1
Source: Nat Commun. 2020 Jan 21;11:402. doi: 10.1038/s41467-019-13960-2 (PMC6972792; doi:10.1038/s41467-019-13960-2)
Supplement: Supplementary file 7 — Source data [file 41467_2019_13960_MOESM7_ESM.zip › Source_Data/FigureS3/FigureS3H_MotifScanOutput/homerResults/motif25.similar.html]

motif25

## Information for motif25

C
G
T
A
C
T
A
G
G
A
T
C
C
G
A
T
T
A
C
G
C
G
T
A
C
T
A
G
A
G
T
C
G
C
A
T
T
A
C
G
G
C
A
T
  
Reverse Opposite:  

C
G
T
A
A
T
G
C
C
G
T
A
T
C
A
G
G
A
T
C
G
C
A
T
A
T
G
C
G
C
T
A
C
T
A
G
G
A
T
C
G
C
A
T
  

|  |  |
| --- | --- |
| p-value: | 1e-34 |
| log p-value: | -7.977e+01 |
| Information Content per bp: | 1.547 |
| Number of Target Sequences with motif | 1216.0 |
| Percentage of Target Sequences with motif | 31.45% |
| Number of Background Sequences with motif | 10042.1 |
| Percentage of Background Sequences with motif | 22.42% |
| Average Position of motif in Targets | 322.8 +/- 326.0bp |
| Average Position of motif in Background | 210.0 +/- 147.7bp |
| Strand Bias (log2 ratio + to - strand density) | 0.0 |
| Multiplicity (# of sites on avg that occur together) | 1.33 |
| Motif File: | file (matrix) reverse opposite |

### Similar de novo motifs found

|  |  |  |  |  |  |  |  |
| --- | --- | --- | --- | --- | --- | --- | --- |
| Rank | Match Score | Redundant Motif | P-value | log P-value | % of Targets | % of Background | Motif file |
| 1 | 0.876 | T C A G C T G A C T A G G A T C G C A T C A T G C T G A C T A G G A T C G C A T C T A G G A C T | 1e-31 | -72.186010 | 5.74% | 2.22% | motif file (matrix) |
| 2 | 0.840 | C T A G A G T C A C G T T A C G C G A T C T A G G T A C G T C A | 1e-27 | -62.861603 | 38.80% | 30.18% | motif file (matrix) |
| 3 | 0.831 | A T C G C T A G C T A G G A T C C G A T A T C G G C T A T C A G G A T C | 1e-25 | -58.513822 | 20.10% | 13.66% | motif file (matrix) |
| 4 | 0.793 | A C T G A G T C C G T A A C T G C T G A A C T G A G T C | 1e-20 | -47.278022 | 38.93% | 31.46% | motif file (matrix) |
| 5 | 0.846 | G C A T G C T A T C G A T A G C C G T A T C A G G T A C G C A T A G T C T C G A C T A G A G T C G C T A | 1e-17 | -39.852364 | 2.02% | 0.58% | motif file (matrix) |
